# An Evolutionary Perspective of Neoplastic Diseases in the Universe

**DOI:** 10.7759/cureus.4030

**Published:** 2019-02-07

**Authors:** German Torres, Sherry M Zakhary, Joerg R Leheste

**Affiliations:** 1 Neurology, Medaille College, Buffalo, USA; 2 Radiology, Mount Sinai Hospital, New York, USA; 3 Epidemiology and Public Health, Minnesota College of Osteopathic Medicine, Gaylord, USA

**Keywords:** cancer, exoplanets, invertebrate organisms, convergent evolution, ionizing radiation

## Abstract

The existence of exoplanets orbiting low mass-stars is one of the most significant discoveries of our time. Especially intriguing to us is the possibility that Earth-sized exoplanets within a habitable zone might harbor life-forms that resemble our own RNA/DNA-based species. We further narrow this theoretical possibility with the following question: if alien life does indeed exist elsewhere, would extraterrestrial life be burdened with earthly diseases? Given that the chemistry of the universe is subject to specific rules, restraints, and predictable outcomes, we argue that cancer-signaling pathways might be programmed into the life cycle of habitable exoplanets. This hypothetical prediction is also based on evolutionary convergence, the repeated emergence of biological similarity that occurs when disparate life-forms adapt to comparable selection pressures. The possibility that mutations and nucleotide base rearrangements that drive cancer growth might be fixed in the chemical hardware of alien life provides us with the opportunity to wonder and consider the origins, evolution, and ubiquity of disease beyond Earth.

## Introduction and background

**Figure 1:**
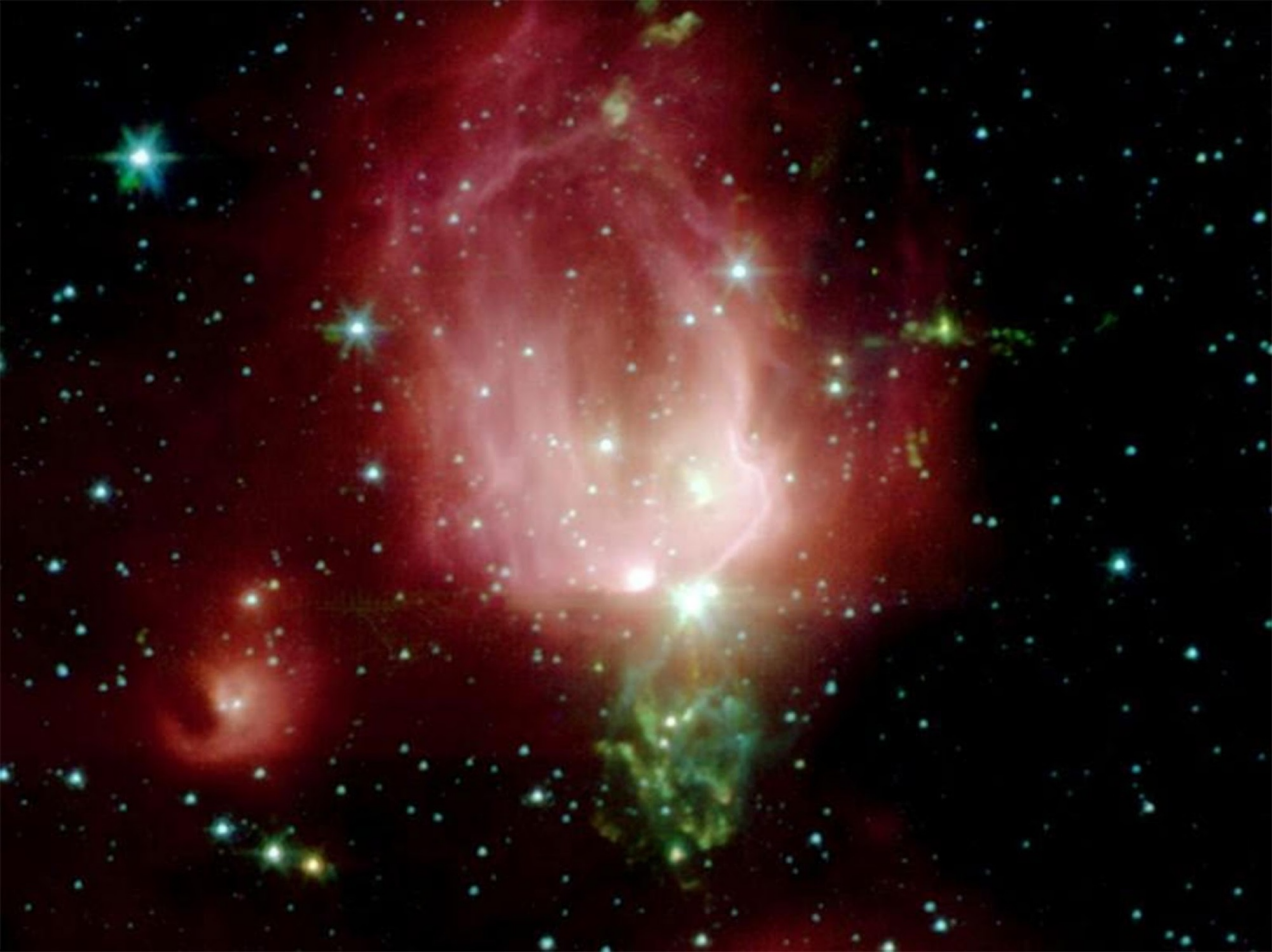
Exoplanets are abundant throughout the observable universe The image depicts a cluster of bright young stars known as NGC 7129 as obtained with NASA’s Spitzer Space Telescope (JPL. NASA. Gov).

The fact that exoplanets transiting stars such as *Proxima Centauri* have been detected light-years from Earth raises an intriguing question: are these distant exoplanets capable of harboring life? Indeed, detection of exoplanets and detection of organic molecules on their surfaces open up a wide range of ideas about ingredients, mechanisms, and environmental pathways required for the evolution, persistence, and maintenance of life. Along the same lines, exoplanetary data also challenge established ideas about our understanding of Earth systems and the origins and early evolution of life on our planet [1]. In this context, more theoretical ideas are needed to provide testable predictions for the occurrence of chemical products on alien life. Specifically intriguing to us is the possibility that alien life might be burdened with Earthly molecular mechanisms (e.g., ligands, receptors, a core set of transcription factors, and cis-regulatory elements) that characterize particular diseases (Figure 1). Of all diseases afflicting life-forms on Earth, cancer is perhaps the most insidious for several reasons: it is a disease widely diagnosed in invertebrate and vertebrate taxa and one that emerged from the recesses of Earth’s most extreme environments close to a billion years ago. If life exists elsewhere, therefore, cancer-signaling pathways would likely be general features of alien life, given the constraints of biological material and the ability of natural selection to favor similar solution outcomes to common environmental problems. That a particular Earthly disease might evolve convergently on exoplanets in response to similar selection pressures would suggest that fundamental life processes are probably universal and that cancer is likely an indistinguishable feature of alien life. 

We question here the scientific possibility of oncogenesis evolving outside Earth now that we know that neoplastic diseases are prevalent in most major invertebrate taxa and that genes linked to cancer span an evolutionary time-frame that stretches back at least hundreds of millions of years. Rather than presenting a litany of pros and cons for the possibility of alien life elsewhere, we focus on two major issues: (i) cancer biology with an emphasis on neoplasia in a number of invertebrate organisms (e.g., marine bivalve mollusks); and (ii) arguing that neoplastic processes, including certain signaling pathways that drive oncogenesis, might be present in disparate exoplanets hosting life. 

## Review

Cancer in invertebrate and vertebrate organisms

**Figure 2:**
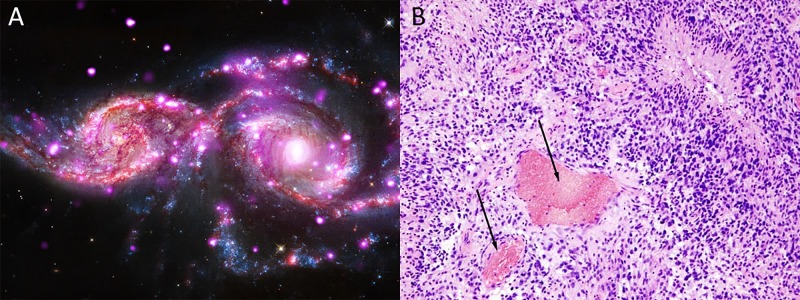
Certain Earthly diseases might be intrinsically associated with alien life; (A) depicts a pair of spiral galaxies (NGC 2207 and IC 2163) similar to that of the Milky Way (NASA/JPL-Caltech.Gov); (B) shows a histological specimen depicting a high-grade glioblastoma (black arrows) of the human cerebral cortex (Cancer Genome Atlas) Magnification 20X for (B)

The prevailing consensus in oncology is that cancer arises when DNA mutations and nucleotide base rearrangements generate genetically distinct cells that target organs in a patchy and asymmetrical manner [2] (Figure 2A, 2B). Thus, cancerous cells somehow subserve the developmental barriers of cell growth and collective migration adopted by epithelial and mesenchymal cells in humans and other vertebrate animals. However, the occurrence of cancer is also observed in a number of invertebrate species distantly related to humans. For instance, cancer-related genes have been identified in a number of species of marine bivalve mollusks (e.g., oysters) [3], and members of the phylum Porifera (e.g., sponges) [4]. Therefore, it is now widely accepted that a significant number of disease-related genes evolved in invertebrate taxa long before the appearance of the vertebrate body plan approximately 530 million years ago [5]. It should be noted that neoplasia in marine bivalves, for instance, follows a similar pattern of disease progression as documented in vertebrate cancers: excessive growth of aberrant cells, dissemination of these cells through circulatory systems and infiltration and seeding of dominant sub-clones into organs [6]. Thus, it appears that cancer growth is usually driven by highly conserved signaling pathways present in extant invertebrate and vertebrate species. From this point of view, organisms with dissimilar genetic backgrounds have evolved chemical signals of similar function *via* different evolutionary routes. This particular process is known as convergent evolution and exemplifies the ability of distant populations to respond predictably to environmental challenges [7-10]. Alternatively, it is also conceivable that cancer-related signaling pathways are critical for a wide variety of cellular processes and therefore have evolved together to maintain a conserved and regulated function. In this latter case, the evolution of phenotypic similarities would be attributed to shared or common ancestry [11-12]. Regardless of how discrete populations use different molecular toolkits to achieve similar functional outcomes, the fact that neoplastic processes are detected in invertebrate and vertebrate animals is of great interest because it strongly suggests that evolution has hit upon the same solution to the same problem time and time again [13]. Moreover, the resemblance of cancer-related signaling pathways between animal taxa raises the possibility of biased genetic constraints owing to similar natural selection pressures. If this proves correct, the likelihood that this Earth-based strategy would be detected elsewhere is relatively high, as both structurally and functionally neoplastic family proteins represent some of the earliest forms of complex chemistry surviving Earth’s most extreme environments. 

Oncogenic proteins in invertebrate organisms

**Figure 3:**
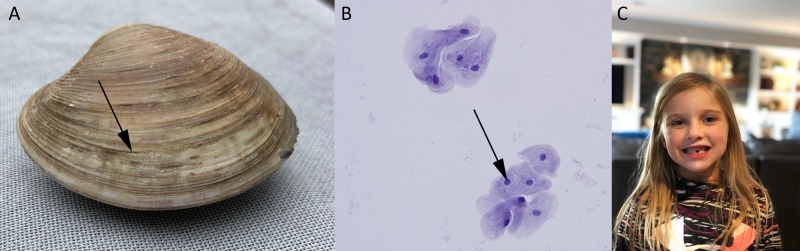
Differences between multicellular (A) invertebrate and (C) vertebrate species are readily apparent in their respective phenotypes; however, differences between multicellular species are counterbalanced by the sequence-based similarities of nucleic acid molecules in their cells; (A) Clam: black arrow points to growth ring; (B) Vertebrate cells: human cheek cells (stratified squamous epithelia) stained with hematoxylin and eosin solutions (black arrow points to the nucleus of a stratified squamous cell); (C) Human Magnification 20X for (B)

Identification of cancer-related signaling pathways in invertebrates provides a way to assess the evolution of molecules linked to human disease [14]. For example, the p53 signaling network suppresses tumor progression in humans, and accordingly, mutations of this network often lead to cancer [15]. Of interest, the p53 protein is expressed in most invertebrate taxa, including sea anemones, clams, mussels and squid [16-17]. Other oncoproteins expressed in invertebrates include nucleoside-diphosphate kinases (Nme/Nm23/NDPK) and mouse double minute (Mdm2 and Mdm4) protein homologs which have been studied in the context of oncogenesis [18-20] (Figure 3A-3C). The prevalence of functionally analogous protein domains and mechanisms leading to tumor formation in mollusks and sponges suggests that the aforementioned proteins originally evolved to trigger apoptosis in damaged cells and to protect germ cells from the deleterious effects of ionizing and/or cosmic radiation. In this context, ionizing radiation (radiation at the high-energy level of the electromagnetic spectrum) is a ubiquitous feature of the universe, having wide-ranging and profound effects on living organisms regardless of biochemistry and genetic background [21-22]. As well known, ionizing radiation damages nucleic acids and proteins, and long-term exposure to solar flares and brightness produces high rates of cancer (e.g., skin cutaneous melanoma). Thus, ionizing radiation is a strong selective pressure with a direct effect on life survival. Because of this, it is likely that all domains of life must adapt to similar selective pressures by evolving analogous signaling pathways that reduce the ability of a tumor cell to survive and proliferate its genome (i.e., to reduce the fitness landscape of tumors). 

Ionizing radiation and oncogenesis

**Figure 4:**
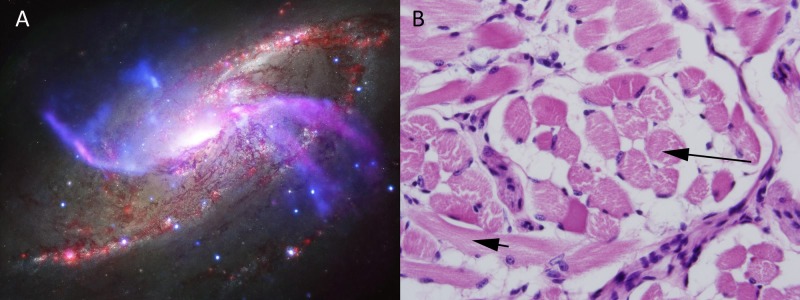
(A) depicts a galaxy known as NGC 4258 with spiral arms glowing in X-ray and optical light emissions (JPL-Caltech. NASA. Gov); (B) depicts a histological specimen of the mouse tongue showing muscle cell layers (muscularis mucosa; short black arrow) and von Ebner’s glands (long black arrow). Magnification 20X for (B) Multi-planetary systems are not static but constantly evolve in response to stellar ionizing radiation. Ionizing radiation is not a stochastic event with a random probability distribution, but rather a deterministic phenomenon in the observable universe.

It should be noted that discussions of alien life being sensitive to ionizing radiation must be based on what we know about life on Earth. Based on our current understanding, exposure to gamma rays, X-rays and the higher ultraviolet part of the electromagnetic spectrum causes DNA double-strand breaks, modifies histone architecture and generates reactive oxygen species that trigger genetic errors within somatic cells [23-27]. Theoretically, radiation stressors in the range of 290-320 nm (and far ultraviolet radiation, 180-280 nm) would have forced early life forms to evolve protective chemical and metabolic strategies against parts of the electromagnetic spectrum. Accordingly, lipid membranes, myelin phospholipids, calcium carbonate-containing shells, melanic scales and feathers and much later the outer skin layers of multicellular organisms would have been most affected by electromagnetic radiation and the most likely burdened with tumor formation. This theoretical thinking resonates with and informs major themes in cancer biology. For example, under this scenario, cancer of body surfaces (mucosal sites) would have been the “first” oncogenic event associated with a defined exposure to a carcinogen: ionizing radiation (Figure 4A, 4B). Additional cancers would have been derivatives from this “first” oncogenic event, including cancers associated with metabolism (e.g., pancreatic and kidney cancer) and cancers associated with the release of burned material such as polycyclic aromatic hydrocarbons (e.g., lung cancer). In this context, normal skin cells harbor a large degree of mutations, and accordingly, skin cutaneous melanoma has the highest mutational load among 23 human cancer types [28]. This may reflect the long evolutionary trajectory this cancer type has endured, including its interplay with ancient environmental pressures, development of cell-specific fitness and transitions from normal to malignant cell function. 

Cancer as a characteristic feature of alien life 

Our hypothesis that life-forms elsewhere would have features resembling Earthly diseases such as cancer is based partly upon the following observations: (i) Chemical elements used for life such as carbon, liquid water, nitrogen, phosphorus, sodium and other array of elements (e.g., sulfur and calcium) all linked by the unceasing flow of electrons and protons are widespread in the universe [29-32]. In addition, amino acids (the building blocks of proteins), constituents of lipids and soluble carbohydrates (i.e., sugars) have been found in meteoritic material [33-35]. These data point to the universality of atomic elements from which signaling pathways can be built. (ii) Chemistry all the way as far as we can see. The cycles and reaction chains of chemical products that give rise to signaling pathways are not random events but rather follow deterministic states. For example, atoms form chemical bonds (e.g., ionic, covalent, polar and non-polar covalent bonds and van der Waals forces) that undergo specific and distinct arrangements to achieve lower potential energies and separation of charge. All of these chemical properties limit or constrain the function of atoms or groups of atoms on Earth and elsewhere in the universe as far as we can see. (iii) As previously stated, evolutionary studies indicate that p53 and Mdm2 genes predated the appearance of cancer. Thus, it is thought that these genes evolved from unrelated functions to later assume a signaling role in preventing the formation of tumors. The p53 family of genes including, p63 and p73 are ancient DNA sequences with the earliest indication of function dating back to sea anemones (e.g., *Nematostella vectensis*), roundworms (e.g., *Caenorhabditis elegans*) and insects (e.g., Drosophila melanogaster) over a billion years ago [16,36]. Interestingly, ancestral p53/p63/p73 genes might have already been present even in single-cell organisms such as choanoflagellates, the closest relatives of sponges thought to have appeared during the late Precambrian era ~2.5 billion years ago [16,37]. The reconstruction of these genes and their long evolutionary history suggests that p53/p63/p73 family of genes evolved early to protect their DNA blueprint from stellar ionizing radiation and probably from other environmental stressors of that long gone era [38]. The functional conservation of this property allows for some predictions on when cancer first emerged on Earth. Based upon the aforementioned data, it is likely that cancer is an ancient evolutionary innovation co-opted to evolve along group traits during the transition between unicellular and multicellular life over 600 million years ago [39]. Indeed, several lines of evidence suggest that cancer is a breakdown of multicellularity, in which gene networks revert back to a unicellular life by undoing the molecular constraints of cell-cell adhesion, cell motility and cell replication [40-43]. If this suggestion is sound, it implies that cancer is embedded in multicellular life, spontaneously arising during consecutive bouts of cell replication across different anatomical sites in a highly non-linear manner [44]. In summary, the lesson to be learned from these insights is that the elemental composition of life is universal at the atomic and molecular level [45]. The signaling pathways associated with malignant cell growth have maintained their functional activities for over one billion years of evolution. To illustrate this latter point, there is very little difference between a clam developing leukemia and a human diagnosed with acute myeloid leukemia as both metazoan organisms show aberrant over-expression of the p53 protein [46-50]. Lastly, the transition from unicellular to multicellular life is thought to have unmasked an ancestral molecular signal which periodically reveals itself by altering a gene’s internal code; an evolutionary event that has permanently sent multicellular organisms down the road of a cancer-riddle existence. 

Against this background, is the commonality of cancer on Earth a sign that neoplasia might also be present in exoplanets harboring life? As previously argued, constraints on chemical and biological materials and exposure to similar selective regimens will produce widespread convergent phenotypic outcomes. Based upon of what we know on Earth, the immediate answer to the above question is a tentative yes: The chemistry and biology of alien life will most likely be the same as that of Earth, because there is only one way to assemble living matter and evolution will ensure that life on exoplanets finds that way. Moreover, the fact that most multicellular organisms have an intrinsic risk factor for cancer (i.e., cancer without disease), suggests that if alien biochemistry is also based on multicellular processes, there will likely be a natural bias for cancer to occur. The idea that cancer would be a feature of alien life is proposed with the intent of transcending the chemistry and biology as we know it and to anticipate the unexpected: the existence of diseases on exoplanets. Here on Earth, from the time there were RNA/DNA-inheriting organisms, life-forms have faced off starvation, predation and infection/disease. One could reasonably hypothesize that if these ecological conditions exist elsewhere, they might eventually lead to the clustering of disease-related molecules within alien genomes. Again, this prediction is based upon the idea that cancer is apparently one of the rare benchmarks in the random path of evolution to have compartmentalized in the biochemical architecture of otherwise separate life-forms. And cancer-signaling pathways most likely evolved during the advent of multicellularity to yield new biochemical mechanisms for energy acquisition and/or generation of biomass.

How does this information affect our perspective of disease(s) in the universe? Infection and disease clearly evolved early and often, which is encouraging for the occurrence of cancer elsewhere. Although this prediction is conjectural, we should nevertheless start thinking about the likelihood of disease in the greater universe, its pervasiveness on exoplanets and its impact on extraterrestrial living systems. If diseases (e.g., cancer) are inevitable and pervasive throughout the universe, can we discern the medical history of exoplanets teeming with life? If an alien life has an infection or disease or other medical affliction that modifies its behavior, can we notice their effects? If the disease is indeed common throughout the observable universe, does disease have a single unique origin? If cancer leaps to exist elsewhere in the universe due to its evolutionary and biochemical underpinnings, what about other Earthly diseases with strikingly similar and overlapping regulatory mechanisms (e.g., amyloidosis)? And more importantly, if diseases are signatures of alien life, can they lead to a better understanding of Earthly diseases with the potential for new therapies and improved outcomes?

## Conclusions

In summary, the universality of cancer is attributed to the lifetime exposure of planets to damaging stellar radiation and perhaps to other geochemical conditions that predate multicellular life. We propose that oncogenic proteins will be risky features of alien life with the capacity to evolve early and be driven by convergent signaling pathways that follow the same general principles as our own RNA/DNA-based species. 
